# Reliability analysis using Limit equilibrium and Smoothed Particle Hydrodynamics-based method for homogeneous soil slopes

**DOI:** 10.1371/journal.pone.0300293

**Published:** 2024-03-11

**Authors:** Xuesong Chu, Jiahui Wen, Liang Li

**Affiliations:** School of Civil Engineering, Qingdao University of Technology, Qingdao, P.R. China; Jamia Millia Islamia, INDIA

## Abstract

This paper develops a combined method to predict the volume of sliding mass for homogeneous slopes in an efficient manner. Firstly, the failure surface with minimum factor of safety (FS) in Limit Equilibrium Method is equated to that one determined by Smoothed Particle Hydrodynamics algorithm to obtain the threshold displacement value for unstable and stable particles. Secondly, the threshold displacement value is used to identify the volume of sliding mass using SPH. Finally, a regression model is developed based on a finite number of SPH simulations for homogeneous soil slopes. The proposed LEM-SPH based method is illustrated through a cohesive soil slope. It is concluded that the use of failure surface with minimum FS in LEM tends to underestimate the volume of sliding mass and to give an unconservative risk value. The Coefficient of Variation (Cov) of volume of sliding mass are 0.14, 0.28, 0.4, 0.48, 0.53 for Cov of soil properties = 0.2, 0.3, 0.4, 0.5, and 0.6, respectively. The uncertainty of soil properties has a significant effect on the mean value of volume of sliding mass and therefore the landslide risk value. The proposed method is necessitated for cases where large uncertainties in soil properties exist.

## 1 Introduction

Slope failure (i.e., landslide) is well acknowledged to be one of essential sources of disasters in geotechnical and geological engineering. It is, therefore, of importance to evaluate the stability of a slope, and to quantify the landslide risk. The slope stability problem, as one of three conventional geotechnical problems, has been well understood and the stability level (usually, a factor of safety, FS) of a slope can be obtained by limit equilibrium method (LEM) and strength reduction method (SRM). The FS value characterizes the distance of the current slope safety state from the safety margin (i.e., FS = 1), however, it represents limited information regarding the landslide risk. In order to properly clarify the risk issue, many researchers have performed landslide risk analysis [1–11]. In previous research, the slope failure consequence is simply described by the volume of sliding mass for the failure surface. [1, 4, 5]. The failure surface is usually determined to be the possible slip surface with the minimum FS value in LEM and it can be automatically identified in SRM [12–14] using different contour of shear strain. However, it must be noted that the failure surface with minimum FS in LEM or SRM pertains only to the initial sliding mass due to the inherent postulation in LEM and the difficulty in simulating large displacements in SRM. As a result, the failure surface with minimum FS determined by LEM or SRM is named as initial failure surface in this study. The use of initial failure surface tends to underestimate the slope failure consequence considering that a larger volume of sliding mass represents a larger failure consequence, and vice versa. In order to rationally quantify the slope failure consequence, the full failure surface including the initial failure surface and the subsequent sliding zones may be identified in risk analysis using algorithms pertinent to large deformations such as Material Point Method (MPM) and Smoothed Particle Hydrodynamics (SPH).

MPM, in essence, the extension of Finite Element Method, utilizes Lagrange particles and Euler grids to solve large deformation problems. MPM is widely used to model the post-failure of slopes in geotechnical engineering [15]. The strength reduction method is combined with MPM to conduct slope stability analysis considering depth coefficient [16]. Recently, the MPM is used to simulate the e influences of the content, the size, and morphology of rock blocks on the failure mechanisms for the soil-rock mixture slopes [17, 18]. Liu et al. [19] utilized the MPM to investigate the nonstationarity of random field of soil strength on the post-failure mechanism of slope failures. SPH is originated in astrophysics [20, 21], hydrodynamics and aeronautics [22] and it has been extended to geotechnical engineering [23–30] since an elastic–perfectly plastic soil constitutive model wherein the Drucker–Prager model with associated and non-associated plastic flow rules has been implemented into the SPH formulation [31]. Recently, Herschel–Bulkley–Papanastasiou (HBP) rheological model has been incorporated into SPH and the dynamic catastrophic process of slope sliding after instability is fully revealed [32]. An improved stress normalization algorithm is developed to eliminate the short-scale noise problem in SPH and a subsequent automatic strength reduction factor method is presented to evaluate the factor of safety using SPH [33]. A new GPU-based SPH runout model is developed and applied to revisit the Johnsons Landing Landslide elucidating the phenomenon of superelevation, channel avulsion, and branching flow [34]. Based on the literature review on MPM and SPH, they allow for simulating the post-failure of slopes in terms of run out distance, trails, and threaten to structures. However, to quantify the failure consequence in SPH or MPM in a theoretical manner remains an open question. Following the previous research of our group, SPH is adopted to quantify the slope failure consequence from the aspect of full failure surface.

Since the determination of full failure surface in SPH requires a threshold displacement value to distinguish sliding particles and stable ones, LEM is used to calculate the volume of sliding mass of the initial failure surface for a slope at critical state (i.e., with minimum FS = 1), which can be used as a benchmark in SPH to calibrate a threshold displacement value. Thus, SPH is combined with LEM in the current study to perform landslide risk analysis combining advantage of LEM in finding failure samples in an efficient manner and that of SPH in properly quantifying the failure consequence. This paper starts with description of SPH-based approach to identify the full failure surface and the empirical relation between FS value and Volume of sliding mass. Then, the proposed method is illustrated using cohesive slopes. Finally, conclusions and discussions are made to provide insights into landslide risk analysis.

## 2 SPH-based method for landslide risk assessment

### 2.1 Self-developed SPH program

SPH serves as an alternative and effective method to simulate the whole process of a slope failure including the initiation, propagation, and deposition of sliding mass and to locate the full failure surface for landslide risk assessment. As a mesh-free algorithm, the computation domain is discretized into a finite number of particles, among of which, the interactions are modeled through a kernel function, *W*(.) within an influencing domain defined by a smoothing length *h*. The motion of a particle in SPH is governed by the equations of mass and momentum conservation [22]. The elastic–perfectly plastic soil constitutive model combined with the Drucker–Prager yielding criterion with non-associated plastic flow rules, developed by Bui et al. [31] is adopted. A cubic spline function is used for *W*(.) The Verlet time integration scheme [24, 35] is used for the simulations along the time steps in SPH. To ensure the stability of numerical integration, incremental time step must be limited to an allowable value as suggested by Bui et al. [31]. A Fortran-based SPH program is developed by the corresponding author and it has been validated by large scale slope model failure test induced by rapid drawdown of water [26], and the dry granular flow experiment reported by Hungr [36] and Fei Tsui Road landslide in Hong Kong [27]. The validated SPH program is extended to conduct the current study. Since the aim of this study is to demonstrate an equivalent procedure to determine the threshold displacement value in SPH, more details and fundamentals regarding the SPH are neglected.

### 2.2 Determination of threshold displacement value in SPH

A threshold displacement value is necessary to quantify the full failure surface using SPH. If the threshold value is specified, the particles with larger displacements than threshold value form the assembly of sliding particles and the boundary surface between the assembly of sliding particles and that of stable ones will be the full failure surface in SPH. In order to determine the threshold displacement value, the slope at the critical state (i.e., the failure surface with minimum FS = 1) is solved by SRM or LEM to obtain the initial failure surface. Thereafter, the slope is reanalyzed by SPH. A threshold displacement value is determined by equating the volume of sliding mass of the initial failure surface in LEM or SRM to that one in SPH. It is noted that the initial failure surface can be determined by either LEM or SRM. LEM with circular failure surface is adopted in this study for illustration since circular failure surface is relatively simple to be implemented and it can lead to comparable results with noncircular failure surface assumption for slope cases without significant controlling sliding surface [37]. It is noted that the critical state of the studied slope must be guaranteed. An empirical procedure can be adopted to arrive at the critical state. The FS is firstly evaluated using LEM or SRM, and then the cohesion and the tangent value of internal friction angle of soils are simultaneously divided by FS leading to a combination of soil strength parameters, based on which the critical state of the slope is reached. That is, if this combination of soil strength parameters is taken as the original value for the slope, the minimum FS of the slope is found to be one and the corresponding failure surface is the initial failure surface.

**Fig 1:**
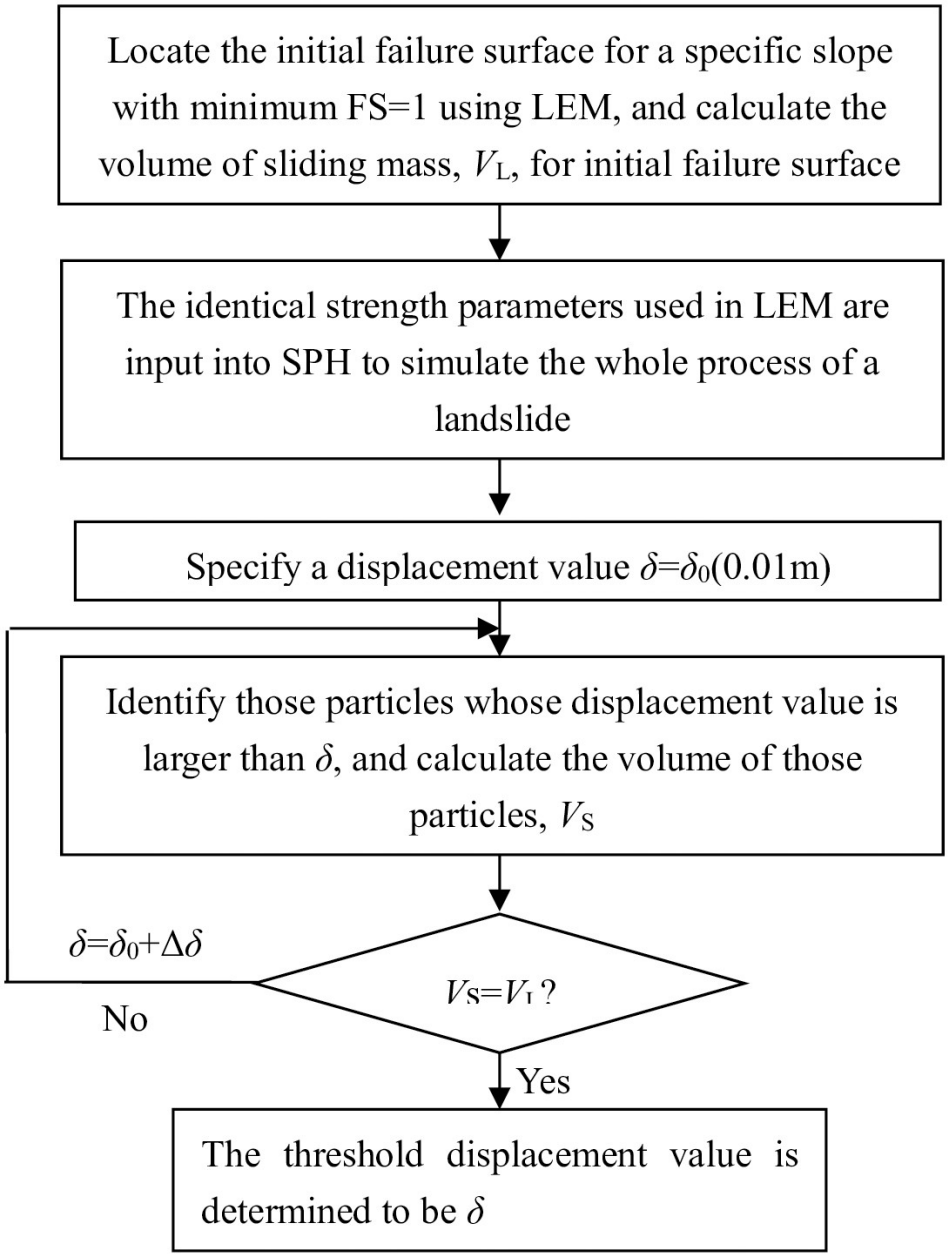
Flowchart of determining the threshold displacement value in SPH.

Fig 1 describes the flowchart for determining the threshold displacement value in SPH. As shown in Fig 1, the failure surface with minimum FS = 1 for a stable slope is firstly obtained by modifying the original values for strength parameters using a trial and error manner. The volume of sliding mass corresponding to the initial failure surface with minimum FS = 1 is calculated to be *V*_L_ and it serve as a benchmark. The strength parameters used in LEM are input into SPH and the slope failure process is modeled obtaining a set of displacement values. A particle is classified into sliding particle or stable one according to its displacement value is larger than or smaller than a specific threshold displacement value *δ*. The summation of all the sliding particles’ volume is the volume of the sliding mass of the full failure surface denoted as *V*_S_. Choice of different threshold displacement values yields different *V*_S_ in SPH. The threshold displacement value leading to *V*_S_ = *V*_L_ is determined to be the expected threshold displacement value. It must be pointed out that there is no rigorous sliding surface in SPH. Based on the previous research output of our group [26–28, 30], the initial failure surface in LEM for a slope at the critical state compares well with the sliding zones denoted by displacement contour in SPH results. The determination of threshold displacement value aims to facilitate the calculation of sliding volume in SPH.

### 2.3 Landslide risk assessment using SPH

Landslide risk assessment requires the information on two aspects, i.e., probability and consequence of a landslide. The landslide probability can be easily calculated using Monte Carlo Simulation (MCS), which has been widely used in slope reliability analysis [1–3, 5]. Recent advancement in MCS has been reported including but not limited to the subset simulation [4] and machine learning aided MCS [38]. The advanced MCS provides an effective tool for conducting landslide risk assessment. It must be noted that the quantification of failure consequence is a site-specific issue and is very complicated depending on the vulnerability of the structures and residents within the runout distance of slope failure. The quantification of failure consequence using sliding volume is tentative for illustration and further refinement is expected. For simplification, as discussed in Introduction Section, the volume of sliding mass for the full failure surface of a landslide is selected to represent the failure consequence, that is, *V* = *C*.

**Fig 2:**
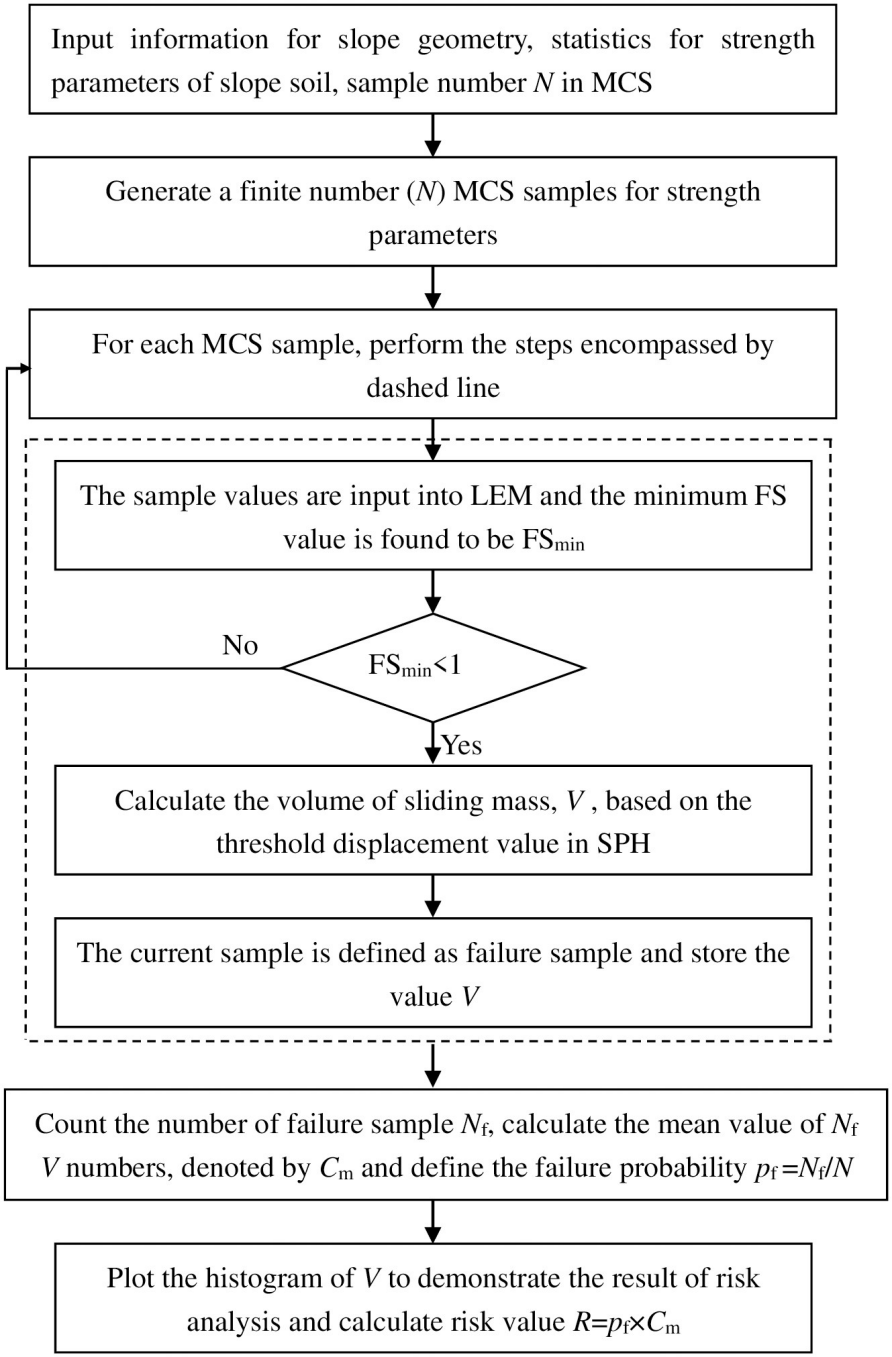
Landslide risk analysis using the proposed method.

Fig 2 demonstrates the flowchart of landslide risk assessment using SPH. In the first step, the necessary information such as slope geometry, statistics of soil parameters, and the total number of MCS samples is provided. Then, a finite number (e.g., *N*) samples following specified distributions are generated. The *i*^th^ set of samples are taken as deterministic inputs in LEM and the minimum FS denoted by FS_min_ is based on to check whether the slope fails or not. If FS_min_ is lower than 1, landslide occurs, and the calibrated threshold displacement value *δ* is used to locate the volume of sliding mass (denoted by *V*) for the full failure surface in SPH. The set of samples leading to FS_min_ <1 is defined as failure sample. In the final step, the total number of failure samples is *N*_f_ and the landslide probability *p*_f_ is thus the ratio of *N*_f_ to *N*. The mean value for *N*_f_ *V* numbers is calculated as *C*_m_, which represents the mean failure consequence. The landslide risk *R* can be determined as:

$$R=p_{\text{f}}\times C_{\text{m}}$$ (1)

To facilitate the comparison, the traditional method [1, 4] for landslide risk is incorporated in the following case studies. Within the traditional method, the volume of sliding mass for the initial failure surface instead of that for the full failure surface in SPH is used for the failure sample.

## 3 Illustration of the proposed method

The proposed SPH-based method is illustrated through a series of cohesive slopes. In **Determination of threshold displacement value** Section, the threshold displacement value for a given cohesive slope is determined using the flowchart described in Fig 1.

### 3.1 Determination of threshold displacement value

**Fig 3:**
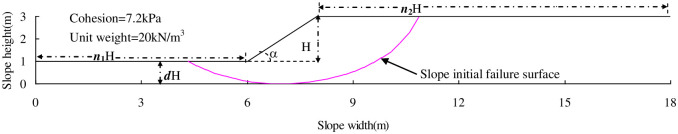
Slope initial failure surface with minimum FS = 1.

**Fig 4:**
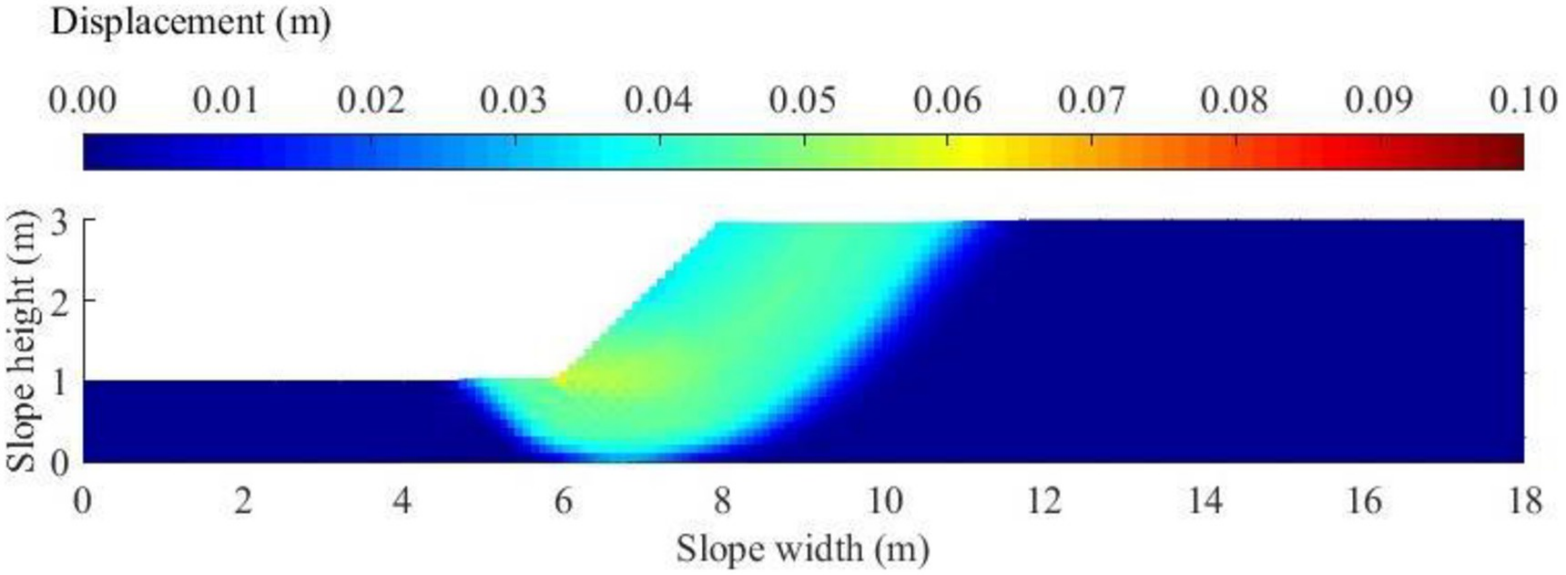
Contour plot of slope displacement at the end of landslide.

**Fig 5:**
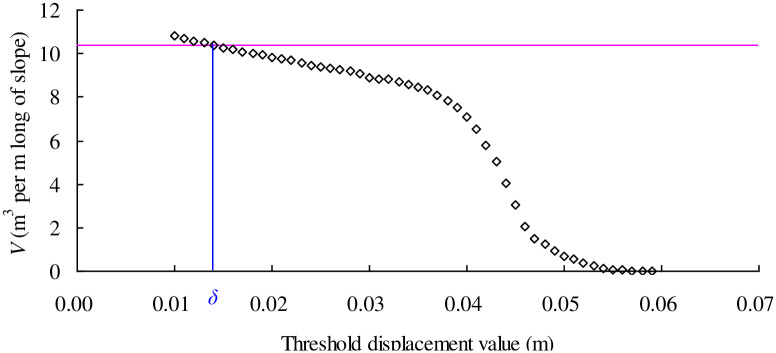
Determination of the threshold value δ for illustrative slope.

As shown in Fig 3, a cohesive slope geometry is described using five parameters: (1) slope height, *H*; (2) slope depth, *dH*; (3) toe width, *n*_1_*H*; (4) crest width, *n*_2_*H*; and (5) slope angle, *α*. A specific cohesive slope with *H* = 2m, *d* = 0.5, *n*_1_ = 3.0, *n*_2_ = 5.0, and α = 45 degree is adopted to demonstrate the determination of the threshold displacement value. The homogeneous soil layer is assumed to have a unit weight = 20kN/m^3^, and cohesion = 7.2kPa. The initial failure surface is calculated by LEM and the results show that the FS for the initial failure surface is 1.0 with a volume of sliding mass = 10.4 m^3^ per m long of slope. The slope domain is discretized into 4010 particles with diameter = 0.1m and the smoothing length, *h*, for all the particles is 0.12m according to the recommendations [25]. The cohesion = 7.2kPa and Unit weight = 20kN/m^3^ are input into the SPH program to simulate the whole process of this cohesive slope and the displacement value for each particle can be obtained after the simulation reaches a steady state. Fig 4 plots the contour of slope displacement and it can be clearly noticed that a series of failure surface occur according to different threshold displacement values. For example, a failure surface emerges between the light blue and dark blue. The full failure surface of the landslide is among these failure surfaces characterized by different colors. In order to determine the threshold displacement value, Fig 5 shows the variation of Volume of sliding mass with different threshold displacement value ranging from 0.01m to 0.07m. As the threshold displacement value increases, the volume of sliding mass decreases. This phenomenon is rational because if a particle with smaller displacement value is regarded as a sliding particle, most part of particles group belongs to sliding particle thereby forming a full failure surface with larger volume of sliding mass. By picking the volume of sliding mass for failure surface in LEM on the variation curve in Fig 5, the threshold displacement value is determined to be 0.014m (see the blue δ on the horizontal coordinate). Therefore, if a particle has a displacement value larger than 0.014m, it will contribute to the volume of sliding mass for the full failure surface in SPH.

Once the threshold displacement value is determined, the landslide risk assessment can be performed following the steps described in Fig 2. It is recognized that SPH simulation of the whole process of landslide needs much time effort compared with the determination of initial failure surface in LEM. Because the volume of sliding mass for the full failure surface is based on the SPH simulation for each set of failure samples (*N*_*f*_ in Fig 2), the work of landslide risk assessment requires demanding computational effort. To improve the computational efficiency, an empirical equation between FS and Volume of sliding mass is developed in the next Section.

### 3.2 Empirical equation between FS and Volume of sliding mass in SPH

**Fig 6:**
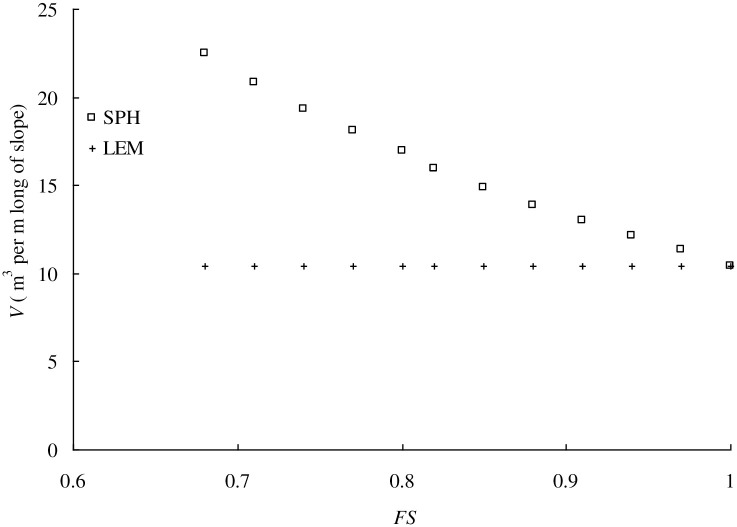
Variation curve between *FS* and Volume of sliding mass, *V*.

Using the same slope geometry shown in Fig 3 and the same SPH model in **Determination of threshold displacement value** Section, a series of cohesion values varying from 5.0 kPa to 7.0kPa sampled at 0.2 intervals are tried in SPH program to simulate the whole failure process at different cohesion values. The volume of sliding mass of full failure surface for each cohesion value is calculated using the threshold displacement value of 0.014m. The variation of volume of sliding mass for full failure surface with FS value is shown in Fig 6. For Comparison, the volume of sliding mass for the initial failure surface determined by LEM is also included in Fig 6. As the FS value decreases, LEM leads to identical *V* values, whereas *V* value increases. This is because LEM only catches the initial failure surface of a landslide but SPH simulates the whole process of a landslide. The difference in *V* values between LEM and SPH increases as the FS decreases and it can be up to 100% for FS = 0.7 case. Considering the failure consequence *C* is equal to the volume of sliding mass, *V*, the smaller volume of sliding mass in LEM underestimates the failure consequence in landslide risk analysis and therefore the SPH-based approach is preferred. Based on the obtained data pairs in Fig 6, a regression equation is developed as:

V=−37FS+46.8 (2)

The volume of sliding mass of the full failure surface of a landslide is calculated by Eq (2) avoiding the time-consuming SPH simulation. It must be noted that the regression equation is only available for the specific slope geometry shown in Fig 3 and it can not be used for other slope geometries (e.g., *H* and/or α changes). A series of homogeneous soil slopes with different geometry parameters (*H*,*α*, *n*_1_, *n*_2_, and *d*) are studied to establish the empirical equation to predict the volume of sliding mass of homogeneous soil slopes.

### 3.3 Empirical equations for homogeneous cohesive soil slopes

Without loss of generality, slope geometries are varied by specifying different sets of parameters of *H*, *d*, *n*_1_, *n*_2_, and α and therefore, a series of unstable cohesive slopes are therefore investigated using the proposed methodology. Table 1 summarizes the details for cohesive slope geometries and soil properties and these slope models are adopted to establish the empirical equations between the FS value and the volume of sliding mass for full failure surface. In Table 1, a combination of *H*, *d*, *n*_1_, *n*_2_, *α*, *c* and FS is defined as one set of slope model. For each set of slope model in Table 1, the threshold displacement value, *δ*, is firstly determined following the steps described in **Determination of threshold displacement value in SPH** Section, and the full failure surface of the corresponding unstable slope is calculated in SPH using this *δ*. Let *V*_L_ denote the volume of sliding mass of the initial failure surface in LEM, and *V*_S_ denote the volume of sliding mass of the full failure surface by SPH, the dimensionless parameter, *ξ*, is defined using the following equation:

$$\xi =\frac{V_{S}-V_{L}}{V_{L}}\times 100\%$$ (3)

**Table 1:** Summary of geometry parameters for cohesive unstable slopes.

Slope ID	*H*(m)	*α*(°)	*n*_1_	*n*_2_	*d*	*c* (kPa)	*FS*
U1	2	45.0	5.0	8.0	0.50	4.0	0.49
U2	3	31.0	3.3	6.7	0.33	5.0	0.49
U3	4	33.7	2.5	6.0	1.00	12.5	0.81
U4	5	26.6	2.0	5.0	0.50	11.7	0.70
U5	8	26.6	2.0	4.0	0.50	20.0	0.75
U6	10	45.0	2.0	5.0	0.50	30.0	0.71
U7	2	45.0	5.0	8.0	0.50	5.0	0.62
U8	3	26.6	3.0	5.0	0.33	5.0	0.53
U9	3	45.0	3.0	6.0	0.33	5.0	0.42
U10	3	36.8	3.3	7.0	0.33	5.0	0.45
U11	4	33.7	2.5	6.0	0.13	10.0	0.77
U12	5	26.6	2.0	5.0	0.50	15.0	0.90
U13	5	26.6	2.0	5.0	0.50	13.0	0.78
U14	6	36.8	2.0	3.3	0.40	20.5	0.90
U15	6	36.8	2.0	3.3	0.40	20.0	0.87
U16	7	41.2	2.0	3.3	0.43	21.0	0.77
U17	7	35.0	2.0	3.0	0.43	22.0	0.86
U18	8	26.6	2.0	4.0	0.50	22.2	0.83
U19	10	45.0	2.0	5.0	0.50	37.5	0.91
U20	10	45	2.0	5.0	0.50	34.61	0.87
U21	2	45.0	3.0	5.0	0.50	7.2	1.00
U22	2	45.0	3.0	5.0	0.50	7.0	0.97
U23	2	45.0	3.0	5.0	0.50	6.8	0.94
U24	2	45.0	3.0	5.0	0.50	6.6	0.91
U25	2	45.0	3.0	5.0	0.50	6.4	0.88
U26	2	45.0	3.0	5.0	0.50	6.2	0.85
U27	2	45.0	3.0	5.0	0.50	6.0	0.82
U28	2	45.0	3.0	5.0	0.50	5.8	0.8
U29	2	45.0	3.0	5.0	0.50	5.6	0.77
U30	2	45.0	3.0	5.0	0.50	5.4	0.74
U31	2	45.0	3.0	5.0	0.50	5.2	0.71
U32	2	45.0	3.0	5.0	0.50	5.0	0.68
U33	4	45.0	3.0	5.0	0.50	13.82	1.00
U34	4	45.0	3.0	5.0	0.50	13	0.94
U35	4	45.0	3.0	5.0	0.50	12	0.867
U36	4	45.0	3.0	5.0	0.50	11	0.80
U37	4	45.0	3.0	5.0	0.50	10	0.72
U38	4	45.0	3.0	5.0	0.50	9	0.65
U39	4	45.0	3.0	5.0	0.50	8	0.58
U40	4	45.0	3.0	5.0	0.5	5	0.36
U41	4	45.0	3.0	5.0	0.5	3	0.22
U42	4	45.0	3.0	5.0	0.5	2	0.15
U43	6	45.0	3.0	5.0	0.50	20.8	1.00
U44	6	45.0	3.0	5.0	0.50	20	0.97
U45	6	45.0	3.0	5.0	0.50	18	0.87
U46	6	45.0	3.0	5.0	0.50	16	0.77
U47	6	45.0	3.0	5.0	0.50	14	0.68
U48	6	45.0	3.0	5.0	0.50	12	0.58
U49	6	45.0	3.0	5.0	0.50	10	0.48
U50	8	45.0	3.0	5.0	0.50	27.64	1.00
U51	8	45.0	3.0	5.0	0.50	27	0.98
U52	8	45.0	3.0	5.0	0.50	25	0.91
U53	8	45.0	3.0	5.0	0.50	23	0.83
U54	8	45.0	3.0	5.0	0.50	21	0.76
U55	8	45.0	3.0	5.0	0.50	19	0.69
U56	8	45.0	3.0	5.0	0.50	17	0.62
U57	8	45.0	3.0	5.0	0.50	15	0.54

**Fig 7:**
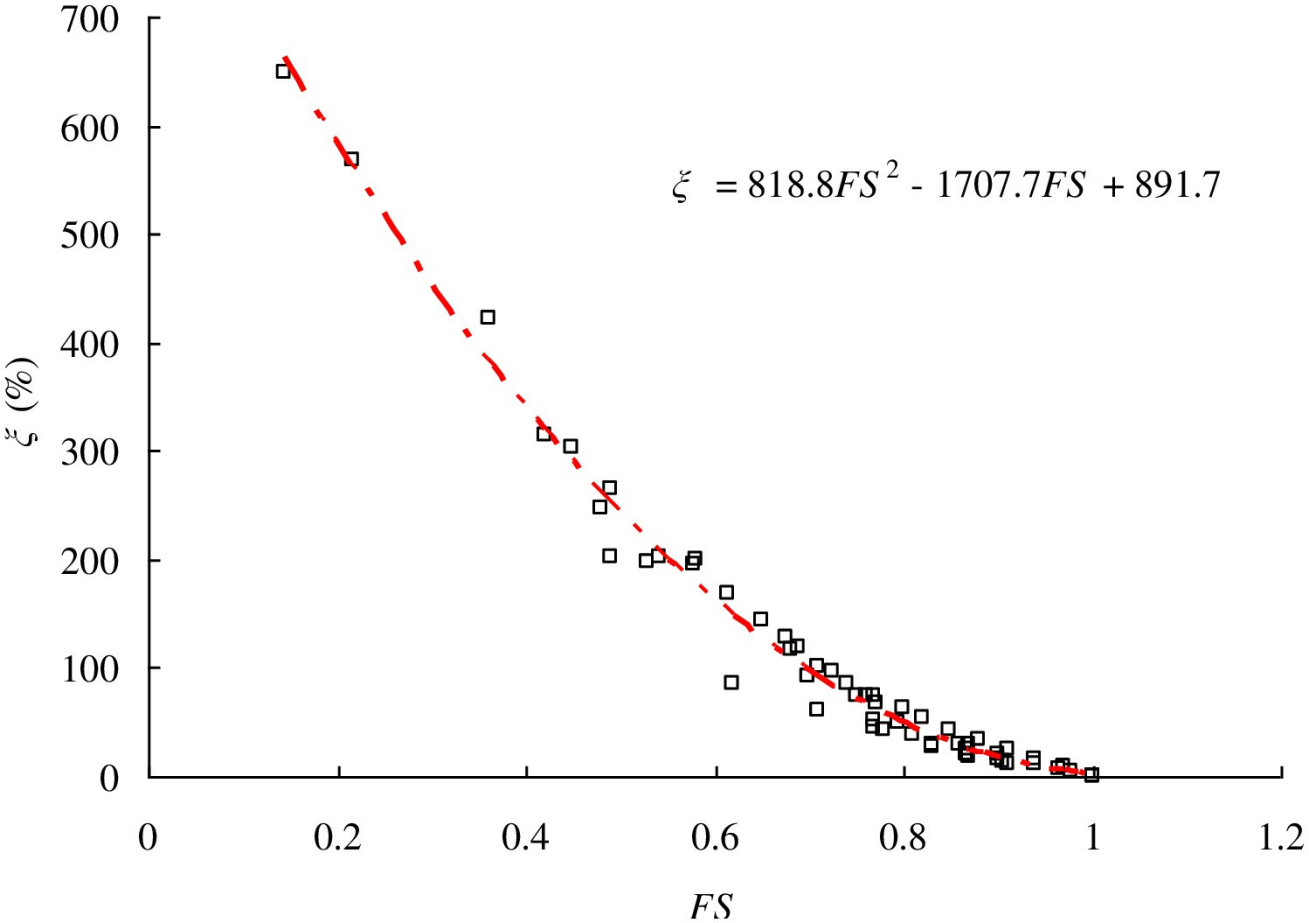
Empirical relationship between FS and *ξ* for a series of cohesive slopes.

Fig 7 plots the variation of *ξ* with FS. As FS increases from 0.2 to 1.0, the value of *ξ* decreases from 600% to almost zero. This phenomenon seems to be rational considering that a smaller FS always implies a more dangerous level of the slope stability leading to severer consequence. To clarify this issue, a second order polynomial regression equation between *ξ* and FS for homogeneous cohesive soil slopes is developed as:

$$\xi =a_{2}FS^{2}+a_{1}FS+a_{0}$$ (4)

where *a*_0_, *a*_1_, and *a*_2_ are constants which should be calibrated based on numerical experiments conducted by SPH and LEM. As Table 1 lists, 57 numerical experiments are performed by SPH and LEM to obtain the Volume of sliding mass for the full failure surface and FS of unstable slopes. The calibrated constants are: *a*_0_ = 818.8, *a*_1_ = -1707.7, and *a*_2_ = 891.7. It must be noted that the number of numerical experiments and the variation of LEM methods will lead to biased regression equation. Readers can add more numerical experiments especially for different *H*, *d*, *n*_1_, *n*_2_, and *α* to improve the resolution of regression equation. Eq (4) is adopted to illustrate the proposed methodology via a cohesive slope which has been studied by Huang et al. [1]. It is noted that the regression in Eq (4) only applies for the single failure mode case observed in homogeneous soil slope. The proposed methodology can not be used directly for homogeneous soil slopes where more than one failure mode is frequently observed.

### 3.4 Application to a cohesive slope after Huang et al. [1]

**Fig 8:**
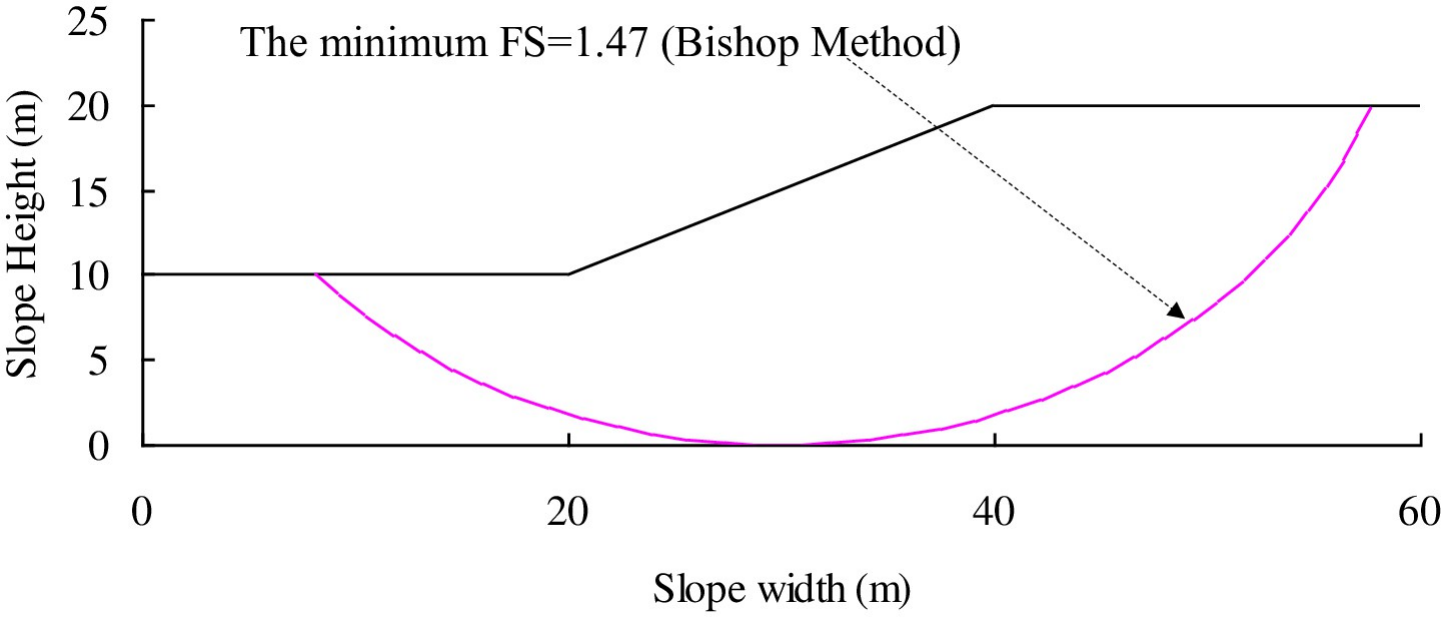
Initial failure surface for the cohesive slope after Huang et al. [1].

A cohesive slope with *H* = 10m, *d* = 1.0, *n*_1_ = 2.0, *n*_2_ = 2.0, and *α* = 26.6° was adopted by Huang et al. [1] to perform risk analysis. The un-drained strength Su is assumed to be log-normally distributed. The mean value of Su is 50kPa, whilst the coefficient of variation (Cov) of Su is 0.5. The soil has a unit weight of 20kN/m^3^. The minimum FS when Su taking mean value is calculated to be 1.47 and the initial failure surface is shown in Fig 8 with volume of sliding mass = 555.4 m^3^ per m long of slope.

It can be easily inferred that the initial failure surface will keep unchanged as the Su value varies and therefore the constant volume of sliding mass(555.4m^3^ per m long of slope) will be adopted to quantify the slope failure consequence *C*, in landslide risk analysis. As described in **Landslide risk assessment using SPH** Section, MCS is adopted to perform landslide risk analysis. The number of MCS samples, *N*, is assumed to be 10,000. To investigate the effect of uncertainty of soil property on the landslide risk assessment, different coefficients of variation (i.e., Cov = 0.2, 0.3, 0.4, 0.5, and 0.6) are assumed with the mean value of Su unchanged. As Table 2 lists, the *p*_f_ at Cov of Su = 0.2 is equal to 3.25%, it increases up to 12.15%, 20.95, 27.74%, and 33.62% at Cov of Su = 0.3, 0.4, 0.5, and 0.6 respectively. The FS values for failure samples are stored in each simulation and they are input into the Eq (4) to obtain the *ξ* values. Finally, the volume of sliding mass for the full failure surface of a landslide is determined.

**Table 2:** Comparison of results between previous method and the proposed method.

	Cov = 0.2		Cov = 0.3		Cov = 0.4		Cov = 0.5		Cov = 0.6	
	M1	M2	M1	M2	M1	M2	M1	M2	M1	M2
*p*_f_	3.25%	3.25%	12.15%	12.15%	20.95%	20.95%	27.74%	27.74%	33.62%	33.62%
*C*_m_	555.4	636.6*	555.4	743.1*	555.4	878.7*	555.4	1038.8*	555.4	1196.5*
*C*_s_	0	86.8	0	206.4	0	350.6	0	497	0	634.0
*R*	18.0	20.7	67.5	90.3	116.4	184.1	154.0	288.2	186.7	402.3

* Note that the mean value of sliding mass for *N*_*f*_ failure samples is adopted to represent the failure consequence for the proposed methodology; M1 = traditional method using the volume of sliding mass of initial failure surface; M2 = the proposed method using Eq (4)

**Fig 9:**
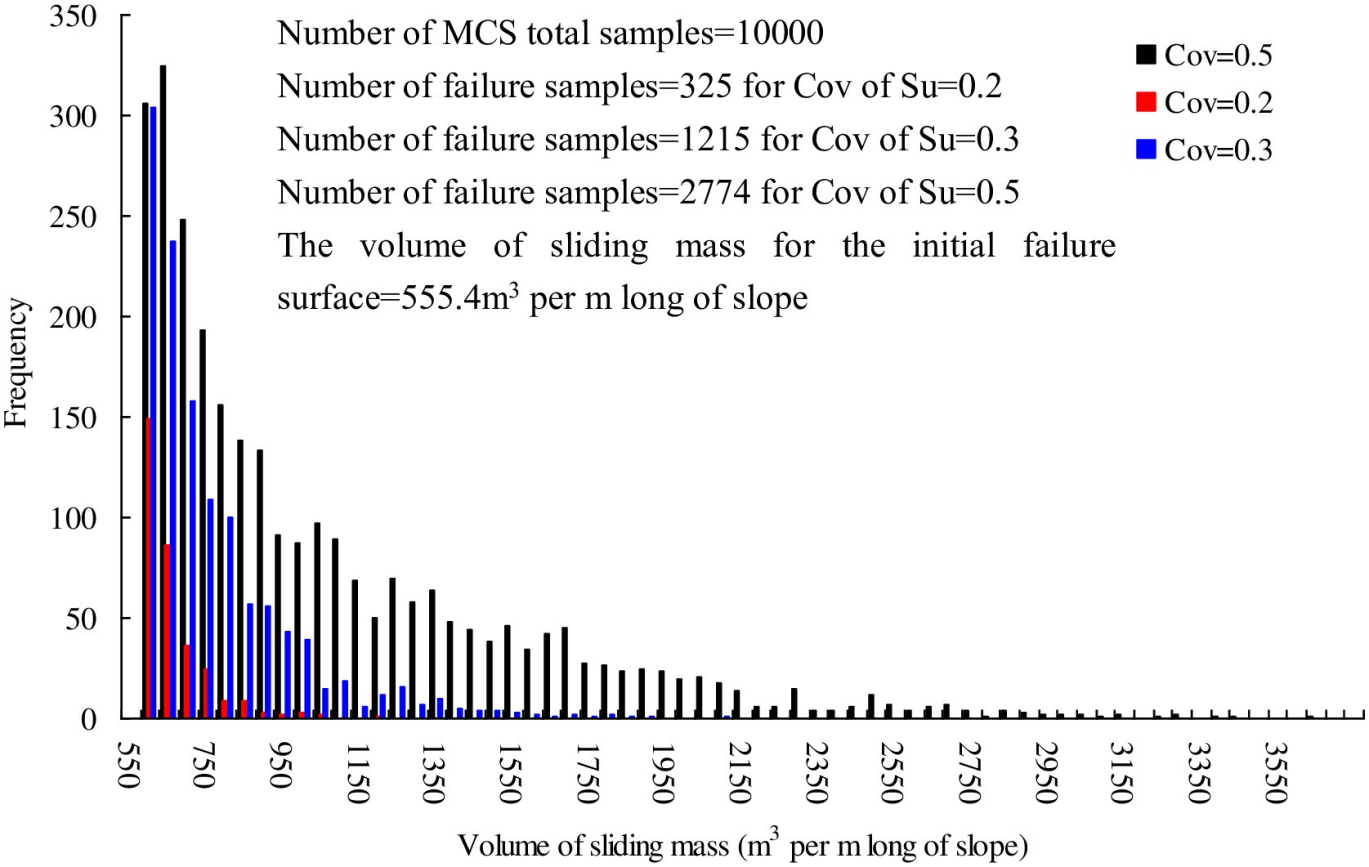
Histogram of volume of sliding mass, *V*, for different Cov of Su.

Fig 9 shows the histogram of volume of sliding mass, *V* for different Cov of Su. For example, at Cov of Su = 0.2, the number of *V* within interval of [550,600], [600,650], [650,700], and [700,750] is 150, 86, 36, and 25, respectively. The number of *V* larger than 750 is rarely small. Similar phenomenon has been observed as Cov of Su increases. As Cov of Su increases, the smaller FS value occurs during Monte Carlo simulation and therefore, a larger value of *V* arises. To properly quantify the influence of Cov of Su on *C*, the mean value (*C*_m_) and standard deviation (*C*_s_) of *N*_f_ *V* numbers are calculated respectively. For example, *C*_m_ = 636.6 and Cs = 86.8 m^3^ per m long of slope at Cov of Su = 0.2 as listed in Table 2. The Cov of *C* (= C_s_/C_m_) is 86.8/636.6 = 0.14. The Covs of *C* are 0.28, 0.4, 0.48, 0.53 for Cov of Su = 0.3, 0.4, 0.5, and 0.6, respectively. It can be found that Cov of *C* is very similar to that of Su. Therefore, the proposed methodology is very useful for those cases where soil properties have significant uncertainties (such as large Cov of Su).

The landslide risk *R* is 18.0, 67.5, 116.4, 154.0 and 186.7 m^3^ per m long of slope at Cov of Su = 0.2, 0.3, 0.4, 0.5 and 0.6, respectively by traditional method (denoted by M1) using the volume of sliding mass for the initial failure surface, while it is 20.7, 90.3, 184.1, 288.2 and 402.3 m^3^ per m long of slope by the proposed method (denoted by M2). This illustrative example demonstrates the necessity of using the proposed method in landslide risk analysis. It is concluded that the use of constant volume of sliding mass for the initial failure surface underestimates the failure consequence of a landslide and leads to a smaller (i.e., unconservative) risk value.

## 4 Conclusions

A new methodology combining SPH and LEM has been developed. Within the new methodology, the widely used LEM is used to identify the slope failure (the minimum FS<1) and the volume of sliding mass for the full failure surface is quantified using SPH-based algorithm. To enhance the computational efficiency of the landslide risk analysis by proposed method, a series of unstable cohesive slopes with different slope geometries and soil properties have been investigated by the proposed methodology to obtain an empirical equation between the volume of sliding mass for full failure surface and its corresponding FS in LEM. The empirical equation is used to quantify the volume of sliding mass, *V*, as the failure consequence, *C*, in case studies. The proposed method balances the computational efficiency and the precision of results. The illustrative example slope is studied by traditional method using initial failure surface and by the proposed empirical equation. The comparison has shown that the use of initial failure surface to quantifying the failure consequence tends to underestimate the failure consequence and to give an unconservative risk value. The uncertainty of soil property has a significant effect on the mean value of failure consequence and therefore the landslide risk value. The Cov of failure consequence, *C*, is very similar to that of undrained strength, Su, for cohesive slope stability. The proposed method is necessitated for cases where large uncertainty in soil property exists. The proposed method can be easily integrated with the MCS to consider the uncertainties of slope soils. As a result, it can be incorporated into the traditional slope reliability analysis. It must be noted that the current research work is limited to homogeneous cohesive slopes and future research may focus on heterogeneous cohesive slopes based on multivariate adaptive regression splines [39, 40].

## Supporting information

S1 Data (ZIP)
